# Relationships between IL-17^+^ Subsets, Tregs and pDCs That Distinguish among SIV Infected Elite Controllers, Low, Medium and High Viral Load Rhesus Macaques

**DOI:** 10.1371/journal.pone.0061264

**Published:** 2013-04-19

**Authors:** Ladawan Khowawisetsut, Kovit Pattanapanyasat, Nattawat Onlamoon, Ann E. Mayne, Dawn M. Little, Francois Villinger, Aftab A. Ansari

**Affiliations:** 1 Department of Pathology and Laboratory Medicine, Emory University School of Medicine, Atlanta, Georgia, United States of America; 2 Department of Immunology, Faculty of Medicine Siriraj Hospital, Mahidol University, Bangkok, Thailand; 3 Office for Research and Development, Faculty of Medicine Siriraj Hospital, Mahidol University, Bangkok, Thailand; 4 Division of Pathology, Yerkes National Primate Research Center, Emory University, Atlanta, Georgia, United States of America; University of Pittsburgh Center for Vaccine Research, United States of America

## Abstract

Comprehensive studies of the frequencies and absolute numbers of the various cell lineages that synthesize IL-17 in the blood and corresponding gastrointestinal (GI) tissues, their correlation with CD4^+^ Tregs, CD8^+^ Tregs, total and IFN-α synthesizing plasmacytoid dendritic cells (pDC) relative to plasma viral load in SIV infection has been lacking. The unique availability of SIV infected rhesus macaques (RM) classified as Elite Controllers (EC), and those with Low, Intermediate and High Viral Loads (HVL) provided a unique opportunity to address this issue. Results of these studies showed that EC demonstrated a remarkable ability to reverse changes that are induced acutely by SIV in the various cell lineages. Highlights of the differences between EC and HVL RM within Gastro-intestinal tissues (GIT) was the maintenance and/or increases in the levels of IL-17 synthesizing CD4, CD8, and NK cells and pDCs associated with slight decreases in the levels of CD4^+^ Tregs and IFN-α synthesizing pDCs in EC as compared with decreases in the levels of IL-17 synthesizing CD4, CD8 and NK cells associated with increases in pDCs and IFN-α synthesizing pDCs in HVL monkeys. A previously underappreciated role for CD8^+^ Tregs was also noted with a moderate increase in ECs but further increases of CD8^+^ Tregs with increasing VL in viremic monkeys. Positive correlations between plasma VL and decreases in the levels of Th17, Tc17, NK-17, CD4^+^ Tregs and increases in the levels of CD8^+^ Tregs, total and IFN-α synthesizing pDCs were also noted. This study also identified 2 additional IL-17^+^ subsets in GIT as CD3^−/^CD8^+^/NKG2a^−^ and CD3^+^/CD8^+^/NKG2a^+^ subsets. Studies also suggest a limited role for IFN-α synthesizing pDCs in chronic immune activation despite persistent up-regulation of ISGs. Finally, elevated persistent innate immune responses appear associated with poor prognosis. These findings provide an initial foundation for markers important to follow for vaccine design.

## Introduction

Dysregulation of the immune response following pathogenic HIV-1 infection in humans and SIV infection in nonhuman primates (NHP) is one of the hallmarks and characteristic of such infections. How such dysregulation is initiated and perpetuated has been a subject of study for the past two decades and continues to be a subject of intense interest. One of the renewed reasons for such interest lies in the finding that in spite of highly effective anti-retroviral therapy (ART) that reduces viral loads to almost undetectable levels for long periods of time still fails to promote the return of immune responses and function to “healthy” levels highlighted by the perpetuation of chronic immune activation and continued increased susceptibilities to infectious agents, accelerated incidence of end organ diseases and neoplasms [1]–[7]. Thus, it seems reasonable to define how such immune responses become abnormal and what strategies can be successfully utilized to reduce and perhaps reverse such events. The NHP model of pathogenic SIV infection provides a powerful tool to begin to unravel the mechanisms involved and to identify the kinetics by which such abnormalities develop and attempt to define more specific targets for immune manipulation.

Our laboratory has been studying the SIV NHP model for some time and data from the outcome of these studies indicate that varying numbers of Indian origin juvenile/adult male rhesus macaques infected intravenously with either SIVmac239 or SIVmac251 following plasma viral load set point achievement, either become ELITE Controllers (EC, <100 viral copies/ml), or maintain Low VL (LVL, <10,000 viral copies/ml), Intermediate VL (IVL, 10,000–100,000 viral copies/ml) or High VL (HVL, >100,000 copies/ml) in the absence of any anti-viral therapy (see Figure S1). Since the VL set point occurs within 4 to 6 weeks post infection prior to the full maturation of an effective acquired virus specific immune response, it is reasonable to assume that events that occur during acute infection, primarily mediated by cells of the innate immune system are likely to play a major initial role in dictating such diverse clinical outcomes [8]–[11].

One of the strategies to identify the mechanisms by which such immune dysfunction is initiated has been the study of the quantitative and qualitative changes in hematopoietic cell lineages post SIV infection. These studies have been based on identifying changes based on ultrastructural studies, cell surface markers unique to each cell lineage, the spectrum of cytokines synthesized and intracellular transcription factors expressed by cells isolated from the peripheral blood, lymph node tissues and jejunal/colo-rectal GI tissues [12]–[21]. Relevant to the studies reported herein, these studies have included the analysis of changes in the frequencies and absolute numbers of IL-17 synthesizing CD4^+^ T cells (Th17), CD8^+^ T cells (Tc17), NK cells (NK-17), regulatory CD4^+^ and CD8^+^T cells (Tregs), interferon-alpha (IFN-α) and tumor necrosis factor alpha (TNF-α) synthesizing plasmacytoid dendritic cells (pDCs) [22]–[33], to name a few. As stated above, these previous studies have focused on either individual or a combination of some of these markers and with samples from a limited number of animals and/or disease stages. This fact prompted us to conduct a detailed study of these sets of markers on tissues from uninfected and SIV infected rhesus macaques with LVL, IVL, HVL and those classified as EC. It was reasoned that a cross sectional study designed to document the relationships between IL-17 synthesizing subsets, the regulatory T cells that potentially influence the function of the IL-17 synthesizing cells and the pDCs in particular the IFN-α synthesizing pDCs would provide initial insights on the role of the IL-17/Treg/pDC axis on the rate of disease progression. Since recent studies have highlighted a prominent role for pDCs particularly during acute infection, a prospective study was subsequently conducted focused on the analysis of IFN-α, its relationship to interferon stimulating genes (ISGs) and TNF-α synthesizing pDCs based on the results obtained on the cross sectional study. Blood and colo-rectal biopsy samples from SIV negative and positive sooty mangabeys were also analyzed in parallel since this species is a natural host of SIV and do not normally develop any detectable disease. Thus, their analysis in parallel was reasoned to provide data that could potentially identify disease resistance similarities in profiles to those obtained on SIV infected rhesus macaques that become EC and/or maintain LVL. Results of these studies constitute the basis of this report.

## Materials and Methods

### Source of Blood and Colo-rectal Biopsy Samples

The Indian origin rhesus macaques (Macaca mulatta) and sooty mangabeys (Cercocebus atys) were all housed at the Yerkes National Primate Research Center (YNPRC) of Emory University (Atlanta, GA) and were maintained according to the guidelines of the Committee on the Care and Use of Laboratory Animals of the Institute of Laboratory Animal Resources, National Research Council and the Department of Health and Human Service guideline titled Guide for the Care and Use of Laboratory Animals. The cross sectional study was conducted on samples from 18 uninfected rhesus macaques (RM), 12 SIV negative sooty mangabeys (SM), 18 naturally infected SM, 55 juvenile/adult male RM that were infected intravenously with either 1000 TCID50 of SIVmac239 or SIVmac251 (grown in day 3 Con-A activated normal rhesus PBMC cultures). The SIV infected RM were retrospectively classified as Elite Controllers (EC, n = 6), RM with LVL (n = 12), RM with IVL (n = 16) and RM with HVL (n = 21) as described above. A subsequent prospective study was performed on PBMC and colo-rectal biopsy tissue samples from groups of rhesus macaques prior to and post IV infection with 1000 TCID50 of SIVmac239 that were a part of another study. The monkeys were retrospectively classified as EC (n = 5), LVL (n = 6), IVL (n = 5) and HVL (n = 6) as outlined above. Plasma viral loads were monitored using bDNA quantitation on aliquots of EDTA plasma by Siemens Inc. (Berkeley, CA).

### Ethics Statement

All animals were born and maintained at the Yerkes National Primate Research Center of Emory University in accordance with the regulations of the Committee on the Care and Use of Laboratory Animal Resources. The animals are fed monkey diet (Purina) supplemented daily with fresh fruit or vegetables and water ad libitum. Additional enrichment is provided and overseen by the Yerkes enrichment staff and animal health is monitored daily by the animal care staff and veterinary personnel, available 24/7. Monkeys showing signs of disease or distress that could not be alleviated using standard analgesics and/or chemotherapy were humanely euthanized using an overdose of barbiturates according to the guidelines of the American Veterinary Medical Association. The studies reported herein were performed under IACUC protocol #2001186 “Innate immunity in SIV infection” which was reviewed and approved by the Emory University IACUC. It has been assigned the IACUC protocol number “YER-2001186-082414GA”. The Yerkes National Primate Research Center has been fully accredited by the Association for Assessment and Accreditation of Laboratory Animal Care International since 1985. All experiments were reviewed and approved by the Emory institutional animal use and care as well as biosafety review Committees.

### Specimen Collection and Mononuclear Cells Isolation from Blood and Rectal Biopsies

Peripheral blood mononuclear cells (PBMC) were isolated from heparinized whole blood using standard Ficoll-Hypaque gradient centrifugation procedure. The mononuclear cells from rectal biopsies were isolated as described previously [26]. Briefly, the biopsies were digested with 500 U/ml collagenase type IV (Worthington, Lakewood, NJ) and 1 U/ml DNase (Promega, Madison, WI) for 2 hours. The biopsy obtained cell suspension was then passed through a 21G needle several times followed by successive passage through a 100-µM followed by a 40 µM cell strainer (BD Falcon™), respectively. The tissue suspension was then overlaid on a 30%/60% Percoll gradient solution (Sigma Aldrich, Saint Louis, MO). After centrifugation at 2,000 rpm for 30 minutes, the mononuclear cells at the interface between 30% and 60% Percoll solution were collected, washed and re-suspended in complete RPMI1640 media. The cell count and cell viability were performed using the trypan blue dye technique. The cell concentrations were adjusted to 1×10^6^ cells/ml in complete RPMI1640 media.

### Fluorochrome-conjugated Monoclonal Antibodies

Various uorochrome-conjugated monoclonal antibodies (mAbs) were used in appropriate combinations to stain the mononuclear cells to determine the frequencies of cell lineages isolated from the blood and rectal biopsies with a focus on lineages that synthesized IL-17 and IFN-α. The mAbs purchased from BD Biosciences (San Jose, CA) included Alexa700-anti-CD3 (clone SP34-2), FITC-anti-CD8 (clone SK1 and clone RPA-T8), PerCP or Pac Blue anti-CD4 (clone L200), FITC-anti-CD14 (clone M5E2), PerCP-Cy5.5-anti-CD16 (clone 3G8), APC-Cy7-anti-CD20 (clone 2H7), PE-Cy7-anti-CD56 (clone NCAM16.2), APC-anti-CD123 (clone 7G3), PerCP-Cy5.5-anti-HLA-DR (clone L243), PE/APC-anti-CD11c (clone S-HCL3) and Alexa700-anti-TNF-α (clone MAb11). The mAb purchased from Beckman Coulter (Brea, CA) included PE-anti-NKG2a (clone Z199), the mAb Alexa647-anti-IL-17A (clone eBio64DEC17) was purchased from eBioscience (San Diego, CA) and the mAb APC-anti-IFN-α (clone LT27∶295) and PE/APC-anti-CD25 (clone 4E3) was purchased from Miltenyi (Auburn, CA).

### Detection of the Frequency of IL-17A Producing Cells by PBMC and Mononuclear Cells from Rectal Biopsies

At least 5×10^5^ mononuclear cells isolated from rectal biopsies and 1×10^6^ PBMC in complete RPMI1640 media were placed in individual well of a 24-well plate and stimulated with 25 ng/ml phorbol-12-myristate-13-acetate (PMA) and 1 µg/ml ionomycin in the presence of 5 µg/ml brefeldin A for 12 hours. After the incubation period, the cells were washed and stained with Live/Dead marker (LIVE/DEAD® Fixable Dead Cell Stain Kits, Invitrogen). The cells were then stained with a panel of cell surface markers at concentrations recommended by the commercial vendor. The cells were then fixed and permeabilized by BD CytoFix/CytoPerm™ solution (BD Bioscience). Cells were washed once with BD Perm/Wash™ buffer (BD Bioscience) and then incubated with anti-IL-17A at 4°C for 30 minutes. The stained cells were washed, resuspended in 1% paraformaldehyde in PBS and kept at 4°C and analyzed by polychromatic flow cytometry using a B-D LSR-II flow cytometer (B-D Immunocytometry, Mountain View, CA). The frequencies of CD4^+^-Th17, CD8^+^-Tc17, and the CD3^−^, CD8^+^, NKG2a^+^ IL-17 synthesizing (NK-17) cells were determined based on the frequencies of IL-17 synthesizing cells on the gated population of total CD4^+^, CD8^+^ and CD3^−^, CD8^+^, NKG2a^+^ cells, respectively as displayed in Figure S2. In the case of the mononuclear cells isolated from the rectal biopsies, we could not utilize the procedure as outlined by Reeves et al (29) because the beads inhibit in vitro cell activation. We therefore expressed the frequencies of IL-17 synthesizing cells based on the frequencies of the parent cell lineage. Thus, the frequencies of CD4^+^-Th17^+^ cells were determined as a percentage of total CD4^+^ T cells and data for the former and the latter are described in each figure that shows data for cells from colo-rectal biopsy tissues. The gating strategy was basically identical to that used for PBMC’s (see Fig. S2). The notation # on the Y axis for each figure signifies the absolute number of cells calculated based on CBC of aliquot of the same sample.

### Detection of the Frequency of IFN-α and TNF-α Producing pDC in PBMC and Mononuclear Cells from Rectal Biopsies

One million mononuclear cells isolated from the rectal biopsies and PBMC were suspended in 1 ml complete RPMI 1640 media and incubated with 3 µg/ml of the TLR7/8 agonist R-848 in the presence of 5 µg/ml brefeldin A for 12 hours. After the incubation period, the cells were washed and stained with Live/Dead marker as described above. In order to identify the pDC population, cells were surface stained with anti-CD14, anti-CD123, anti-HLA-DR, anti-CD20 and anti-CD3. After washing, the stained cells were fixed, permeabilized, and stained with either anti-IFN-α or anti-TNF-α at 4°C for 30 minutes. The stained cells were washed and re-suspended in 1% paraformaldehyde in PBS and kept at 4°C until analysis using standard flow cytometry. Aliquots of the mononuclear cells from the colo-rectal biopsy tissues were also stained for cell surface markers and intracellular expression of IFN-α in parallel without incubation with the TLR7/8 agonist in efforts to determine the frequencies of pDC that constitutively expressed IFN-α.

### Flow Cytometric Acquisition and Data Analysis

Samples were analyzed on a BD FACS LSRII (BD Immunocytometry Systems) and the data obtained analyzed using FlowJo software (Tree Star, Ashland, OR). The compensation setting of BD FACS LSRII was set after acquiring unstained and single-color control samples and calculating the compensation matrix. Data from a minimum of 100,000 events were acquired for the analysis of IL-17 producing cells and at least 300,000 (in 90% of the cases 500,000) events were acquired for the analysis of IFN-α and TNF-α producing pDC.

### Measurements of Interferon Stimulated Genes (ISGs)

One of the outcomes of SIV infection previously documented is the ability of the virus to stimulate the synthesis of a variety of ISGs. In efforts to determine the relative levels of ISGs being synthesized by gastro-intestinal tissues from the various cohorts of animals in this study, we chose to monitor the levels of 2,5 Oligoadenylate synthetase (OAS) and myxovirus resistance protein A (MxA) and for purposes of reference the housekeeping gene GAPDH. The samples analyzed consisted of colo-rectal biopsy tissue obtained mononuclear cells from animals prior to and at days 7, 14, 21, 28 and 56 from 6 uninfected animals, 5 classified as EC, 8 as LVL, 9 as IVL and 10 as HVL animals. The analysis was performed essentially as described by Matzinger et al [34]. RNA was extracted from GI tissue biopsy isolated cells and after treatment with DNase subject to cDNA derivation. The GAPDH, OAS and MxA were run in parallel in the RT-PCR assay [35], [36] and the CT value of the GAPDH subtracted from the values obtained for OAS and MxA (DCT). The values obtained from the baseline (pre-infection) levels (mean of 3 baseline sample value) from the same animal were then subtracted from the values obtained post SIV infection (DDCT) and assuming that the efficiency of amplification of the GAPDH is similar to that of the ISGs the net change in the samples post infection was calculated as 2- DDCT according to the manufacturers instruction.

### Statistical Analysis

All statistical and graphical analysis were performed using the SPSS software (SPSS Inc., Chicago, IL). The non-parametric Mann-Whitney U test was used to compare the data between groups of monkeys. The differences with a p-value less than 0.05 were considered as statistically significant.

## Results

The data presented herein represent in part samples from 55 rhesus macaques (RM) that were infected intravenously with 200 TCID50 of either SIVmac239 or SIVmac251 as described in the methods section. Individual monkeys within this cohort were either classified as EC, monkeys with LVL, IVL or HVL based on continuous monitoring of plasma viral loads as outlined above. All monkeys sero-converted with readily detectable anti-SIV ELISA titers by 6–8 weeks post infection. In each of these analyses, PBMC and cells isolated from colo-rectal biopsy specimens from adult SIV negative and positive sooty mangabeys (SM) were also analyzed for the same markers and included for purposes of comparison. In all cases, data obtained using PBMCs reflect the frequency of the appropriate cell lineage (%) and the absolute numbers of each cell lineage (appearing as Fig. A and B). However, in the case of colo-rectal biopsies, unfortunately we could not utilize the technique previously described by Reeves et al [29] to determine absolute numbers because the addition of the beads inhibited the ability to optimally activate the cells in vitro. In addition, the use of weight of the biopsy material as a relative measure was not found to be reliable with a large variation due to differences in cellular and moisture content. Since the data from the colo-rectal biopsies to a large extent include results on individual subsets of a given cell lineage, data are expressed therefore as the frequency of the subset (in Fig. C for all Figs. except 4, 8 & 9) and in addition to, the frequencies of the parent cell lineage in the same sample (in Fig. D for all except Figs. 4, 8 and 9).

### Marked Decreases in CD4^+^ Th17 Cells Correlates with Plasma Viral Loads

**Figure 1:**
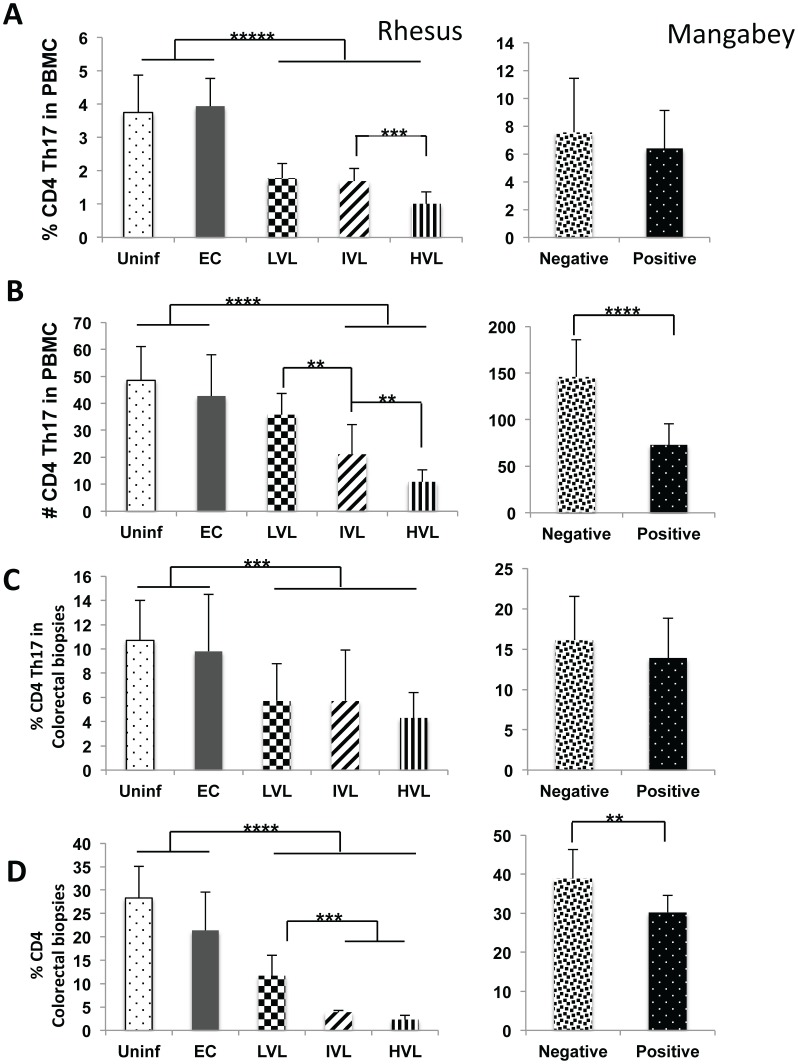
Analysis of Th17^+^ cells in PBMC and corresponding colo-rectal biopsies from SIV infected nonhuman primates. Frequencies (%) and absolute numbers (#) of CD4^+^ Th17 cells in the PBMC and colorectal biopsies of groups of uninfected (Uninf), and SIV infected rhesus macaques classified as Elite Controllers (EC), those with plasma levels of Low Viral Loads (LVL), Intermediate Viral Loads (IVL), and High Viral Loads (HVL) in the left panels. The right panels reflect the same measurements in SIV seronegative (Negative) and SIV seropositive (Positive) sooty mangabeys assayed in parallel. A) Reflects the data on % CD4^+^ Th17 cells in PBMC B) the absolute number of CD4^+^ Th17 cells C) the % CD4^+^ Th17 cells in colorectal biopsy tissues and D) the % CD4^+^ T cells in the same aliquot of biopsies. Statistically significant data are reflected by the number of asterisks with p<0.02 (*). p<0.05 (**), p<0.01 (***), p<0.001 (****) and p<0.0001 (*****) for all figures.

As seen in Fig. 1A, while there was no difference in the frequency and absolute numbers of CD4^+^ Th17 cells in the PBMC from EC as compared with uninfected rhesus macaques, there were clear differences in both the frequency and absolute number of this lineage between EC/uninfected and SIV infected monkeys with LVL, IVL and HVL (p<0.001). In addition, there were differences also in the % CD4 Th17 between IVL and HVL (p<0.01) and as seen in Fig. 1B, the absolute numbers between LVL and IVL and IVL and HVL (p<0.05). Of interest were the finding of a marked decrease in the absolute number but not the frequency of CD4^+^ Th17 cells in the PBMC from SIV positive SM as compared with SIV negative SM (p<0.001). The results for the colo-rectal biopsies were essentially similar to the differences obtained with PBMCs (Fig. 1C depicts % Th17 and Fig. 1D the frequencies of total CD4^+^ T cells). Thus the frequency of CD4^+^ Th17 cells were markedly lower post SIV infection in LVL, IVL and HVL RM (p<0.01) as compared with EC/uninfected controls. These values need to be evaluated in light of the marked decreases noted in the frequencies of total CD4^+^ T cells in the LVL, IVL and HVL animals which decreased down to 2–3% (Fig. 1D) underscoring the profound decrease in IL-17 synthesizing CD4^+^ T cells in the GI tissues. In addition, there was also a significant decrease in the frequency of total CD4^+^ T cells in colo-rectal biopsy tissues from SIV positive SM as compared with SIV negative SM (p<0.05).

### Reduction of CD8^+^Tc17 in Colo-rectal Tissues but not PBMC in SIV Infected RM

**Figure 2:**
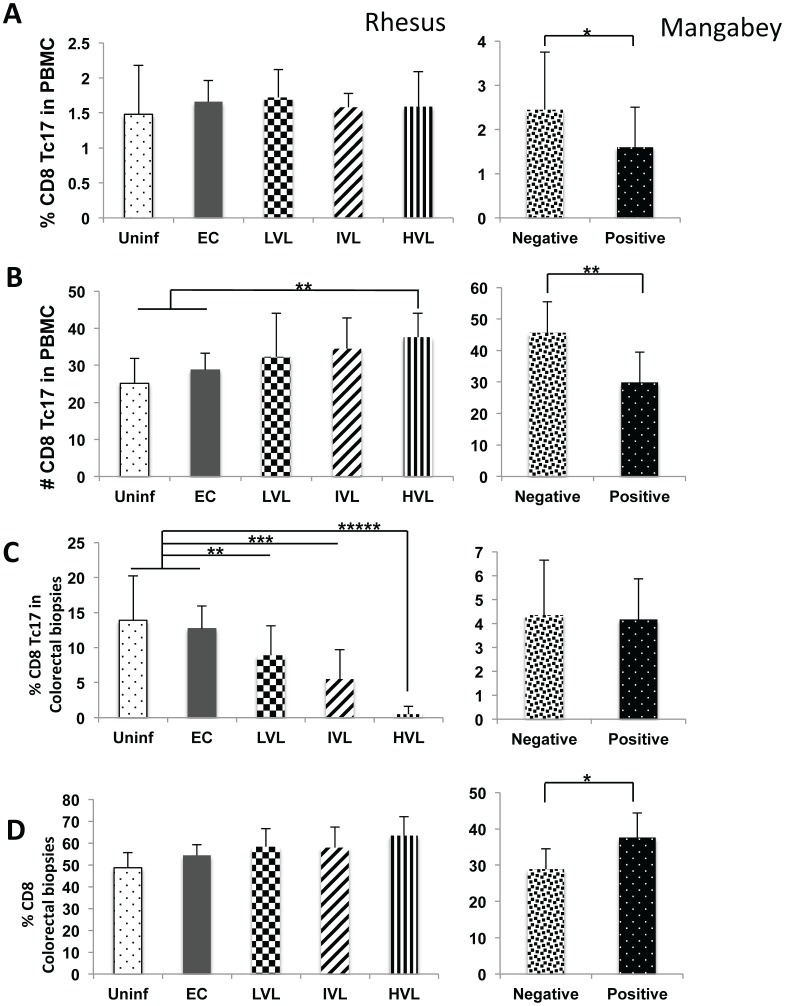
Analysis of Tc17 cells in PBMC and corresponding colo-rectal biopsies from SIV infected nonhuman primates. Nomenclature identical to Fig. 1 except the data reflects values for CD8^+^ IL-17 cells (Tc-17).

While there were no significant differences in the frequencies of Tc17 in the PBMCs of RM in the various groups (Fig. 2A), an increase in the absolute number of total CD8^+^ T cells led to an increase in the absolute number of Tc17 cells specially in monkeys with HVL (p<0.05) (Fig. 2B). Of interest, there was clearly a significant decline in both the frequency (p<0.02) and absolute number (p<0.05) of Tc17 cells in the PBMC of SM post infection. In contrast with the PBMC’s, there was a marked decrease in the Tc17 cells in the colorectal tissues of SIV infected RM and the level of decrease correlated with levels of plasma VL (Fig. 2C), although the number of total CD8^+^ T cells either did not change or showed a slight increase in both the RM and SM post infection (Fig. 2D).

### Marked Changes in the Frequencies and Absolute Number of NK-17^+^ Cells in both the PBMC and Colo-rectal Biopsies

**Figure 3:**
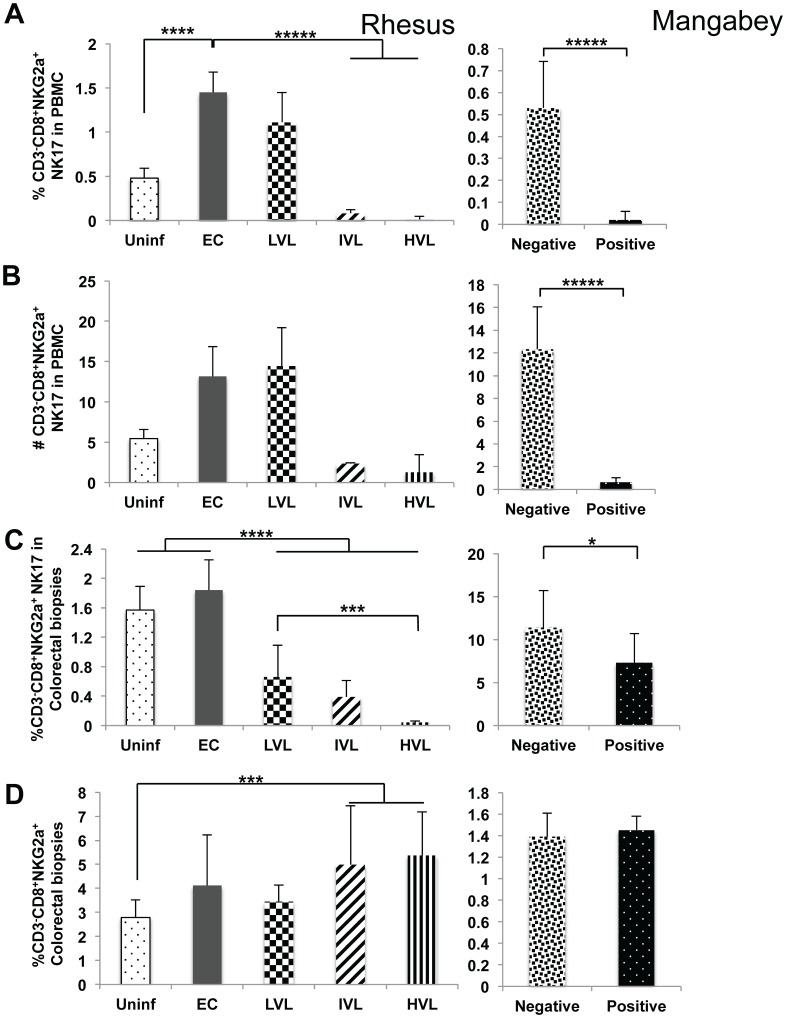
Analysis of NK-17 cells in PBMC and corresponding colo-rectal biopsies from SIV infected nonhuman primates. Nomenclature identical to Fig. 1 except the data reflects values for CD3^−^, CD8^+^, NKG2a^+^ (NK) cells.

Of the cell subsets studied, the frequencies and absolute numbers of NK-17^+^ cells showed the most significant net change with marked increases in the EC and LVL as compared with uninfected RM (p<0.001) and marked decreases in RM with IVL and HVL as compared with EC RM (p<0.0001) as seen in Fig. 3A and 3B. The decrease in this subset of cells, of interest, was also noted in the PBMC of SM post SIV infection in both the frequencies (p<0.0001) and absolute numbers (p<0.01). These NK-17^+^ cells also decreased markedly in frequency in the colo-rectal biopsies tissues from LVL, IVL and HVL SIV infected RM but not EC as compared with uninfected animals even though the frequencies of the NKG2a^+^ NK cells increased suggesting that this decreases in the NK-17^+^ cells is selective and more severe (Fig. 3C & D). There was also a small decrease in the frequencies of NK-17^+^ cells in SIV infected SM as compared with SIV negative SM although the frequencies of total NK cells did not change. Thus, while the decreases in this cell lineage seen in RM with LVL, IVL and HVL appears associated with levels of VL, the significance of the marked increase in EC RM is clearly not related to viremia but likely due to levels of cytokines being released as a consequence of effective viral control. Similarly, the marked decrease of this cell lineage in the PBMC of SIV positive SM as compared with SIV negative SM is difficult to explain and it is also possible that these changes could all be secondary to differences in homing patterns of these cells post infection.

**Figure 4:**
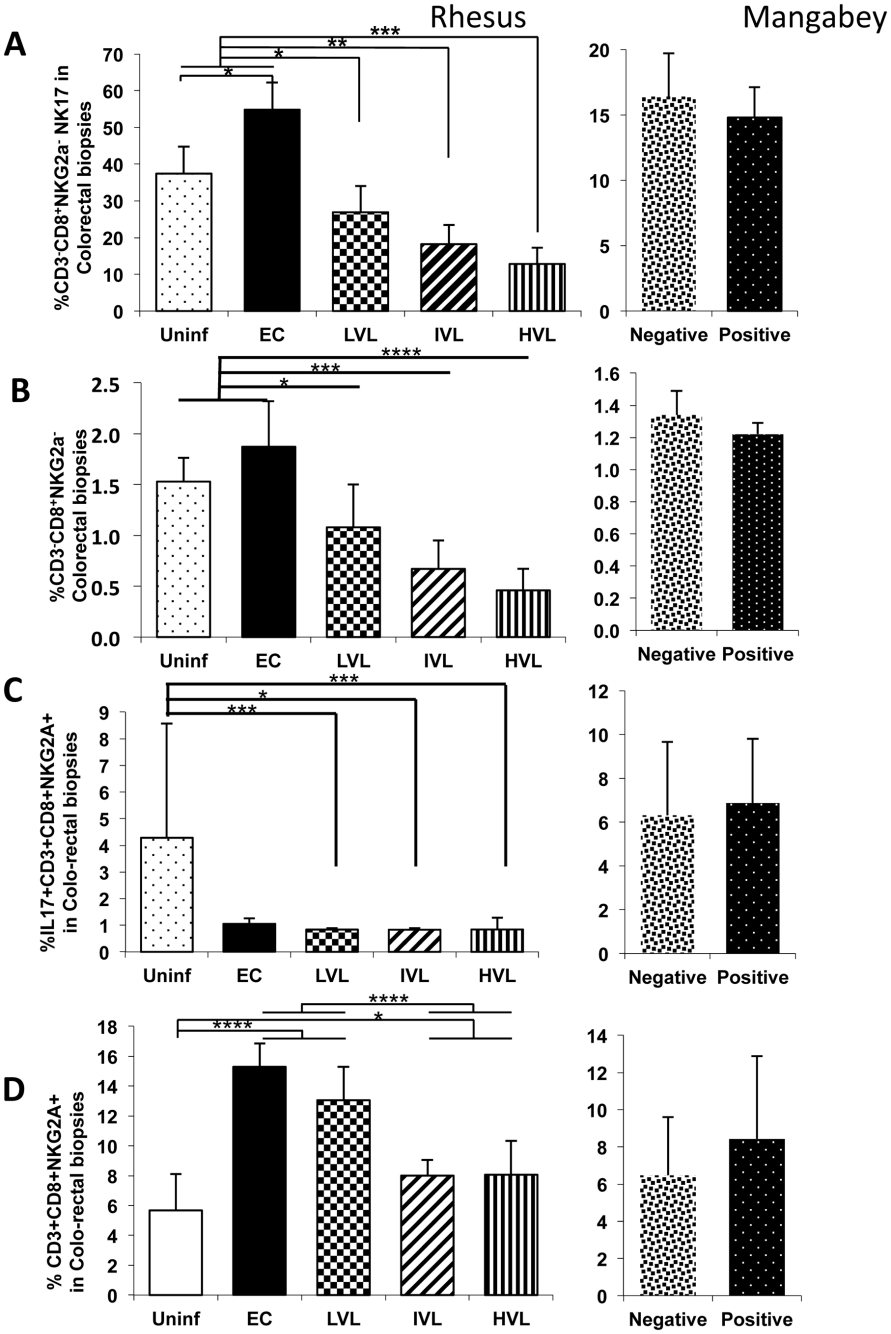
Analysis of novel IL-17^+^ cell subsets cells in colo-rectal biopsies from SIV infected nonhuman primates. Frequencies (%) of CD3^−^, CD8^+^, NKG2a^−^ and the CD3^+^, CD8^+^, NKG2a^+^ subsets of cells in the colo-rectal biopsy tissues from groups of uninfected rhesus macaques, SIV infected rhesus macaques classified as Elite Controllers (EC), those with LVL, IVL and HVL in the left panels. The right panels reflect data on seronegative (Negative) and SIV infected seropositive (Positive) sooty mangabeys. A) Frequencies of the CD3^−^, CD8^+^, NKG2a^−^ subset of NK cells that are IL-17^+^ B) Frequencies of the parent CD3^−^, CD8^+^, NKG2a^−^ cells from the same specimen. C) Frequencies of CD3^+^, CD8^+^, NKG2a^+^ that are IL-17^+^ D) Frequencies of the parent CD3^+^, CD8^+^, NKG2a^+^ cells from the same specimen.

When the frequencies of all the IL-17 synthesizing lineages were being analyzed, it was noted that a significant number of IL-17 synthesizing cells expressed CD3^−^ CD8^+^ NKG2a^−^ phenotype (which includes the NKp44^+^ cells). This subset was only noted in GI biopsy tissues and was thus analyzed. Once again while there was an increase in the frequencies and absolute number of this lineage in EC (p<0.02) compared with uninfected RM (Fig. 4A & B), there was a decrease of this lineage and the fraction that synthesized IL-17 in the LVL, IVL and HVL monkeys and the level of decrease associated with plasma VL. No difference in the frequencies of the parent CD3^−^ CD8^+^ NKG2a^−^ cells and/or the IL-17^+^ fraction of this cell lineage was noted in samples from SM post SIV infection.

### Increases of the Frequencies of a New Subset of CD3^+^ CD8^+^ NKG2a^+^ Cells in Colo-rectal Biopsies of EC and LVL SIV Infected Rhesus Macaques

During the course of the studies on colo-rectal biopsies, it was interesting to find another subset of NKG2a^+^ cells with an unique phenotype some of which also synthesized IL-17 that were not found in detectable levels in the corresponding PBMCs. As seen in Fig. 4D, there appeared to be a significant increase in the frequency of NKG2a^+^ cells that expressed both CD3 and CD8 isolated from the colo-rectal biopsies from each sub-group of SIV infected (EC, LVL and HVL) rhesus macaques. There was no statistical difference between the IVL and LVL. Of interest was the finding that although there is an increase in this subset in tissues from EC and LVL, the frequencies that synthesize IL-17 is markedly reduced in all SIV infected rhesus macaques (Fig. 4C). No detectable change was noted in this subset from SM post SIV (Fig. 4C & D) but it is important to note that a significant number synthesize IL-17 (Fig. 4D). The in vitro functional analysis of an aliquot of this highly purified population of this subset from uninfected RM using flow cytometry revealed that while this subset from rhesus macaques showed no detectable NK cell cytotoxicity, this subset from SIV negative SM showed readily detectable cytotoxicity identifying a significant difference in function between RM and SM (data not shown). A more detailed study of this subset is in progress.

### Decreases in CD4^+^ Tregs in both PBMC and Colo-rectal Biopsies of LVL, IVL and HVL but not EC RM Post SIV Infection

**Figure 5:**
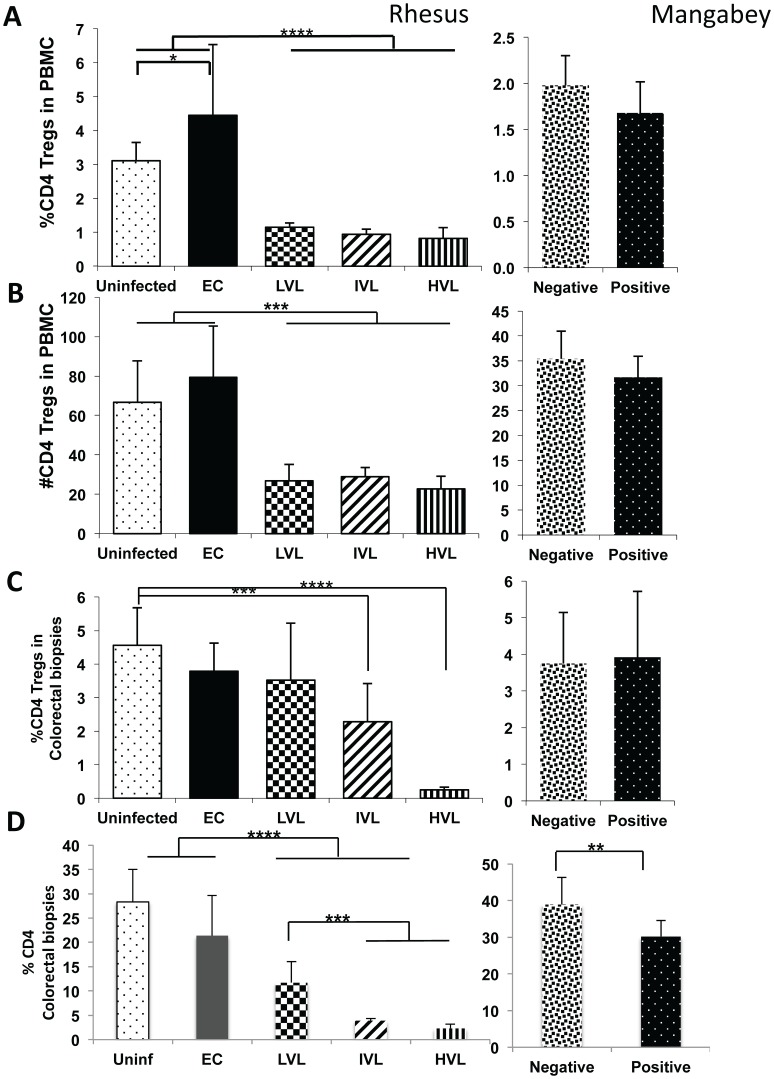
Analysis of CD4^+^ Tregs cells in PBMC and corresponding colo-rectal biopsies from SIV infected nonhuman primates. Nomenclature identical to Fig. 1 except the data reflects values for CD4^+^ Tregs. Please note D) reflects the frequencies of total CD4^+^ T cells in the same sample.

There has been considerable interest in the role of Tregs, in particular, a role in tissues enriched for cells with pro-inflammatory potential such as Th17 and Th1 cells with the view that these Tregs provide a dominant negative regulatory function to minimize local tissue pathology. This cell lineage was thus studied in the same samples from these monkeys. As seen in Fig. 5A and 5B, while there is an increase in the frequencies and, in general, absolute numbers of CD4^+^ Tregs in the PBMC from EC RM, there is a decrease of CD4^+^ Tregs in the PBMC samples from LVL, IVL and HVL RM during chronic SIV infection as compared with the uninfected controls. No difference of this subset was noted in the PBMC samples from SIV infected SM compared with SIV negative SM. When the colo-rectal biopsy tissue samples were examined, while the EC and LVL RM showed variable but statistically non-significant decreases, the IVL and HVL clearly showed significant decreases (Fig. 5C). These decreases though have to be analyzed in the context of decreases in the total CD4^+^ T cells that were also decreased significantly in LVL, IVL and HVL RM (Fig. 5D). The relationships between Tregs and Th17 cells are discussed below. The CD4^+^ Tregs in colo-rectal biopsies from SM did not change in frequency although there did appear to be a significant decrease in the total CD4^+^ T cells (p<0.05).

### Increases in the Frequencies of CD8^+^ Tregs in the PBMC and Colo-rectal Biopsies Post SIV Infection in RM but not SM

**Figure 6:**
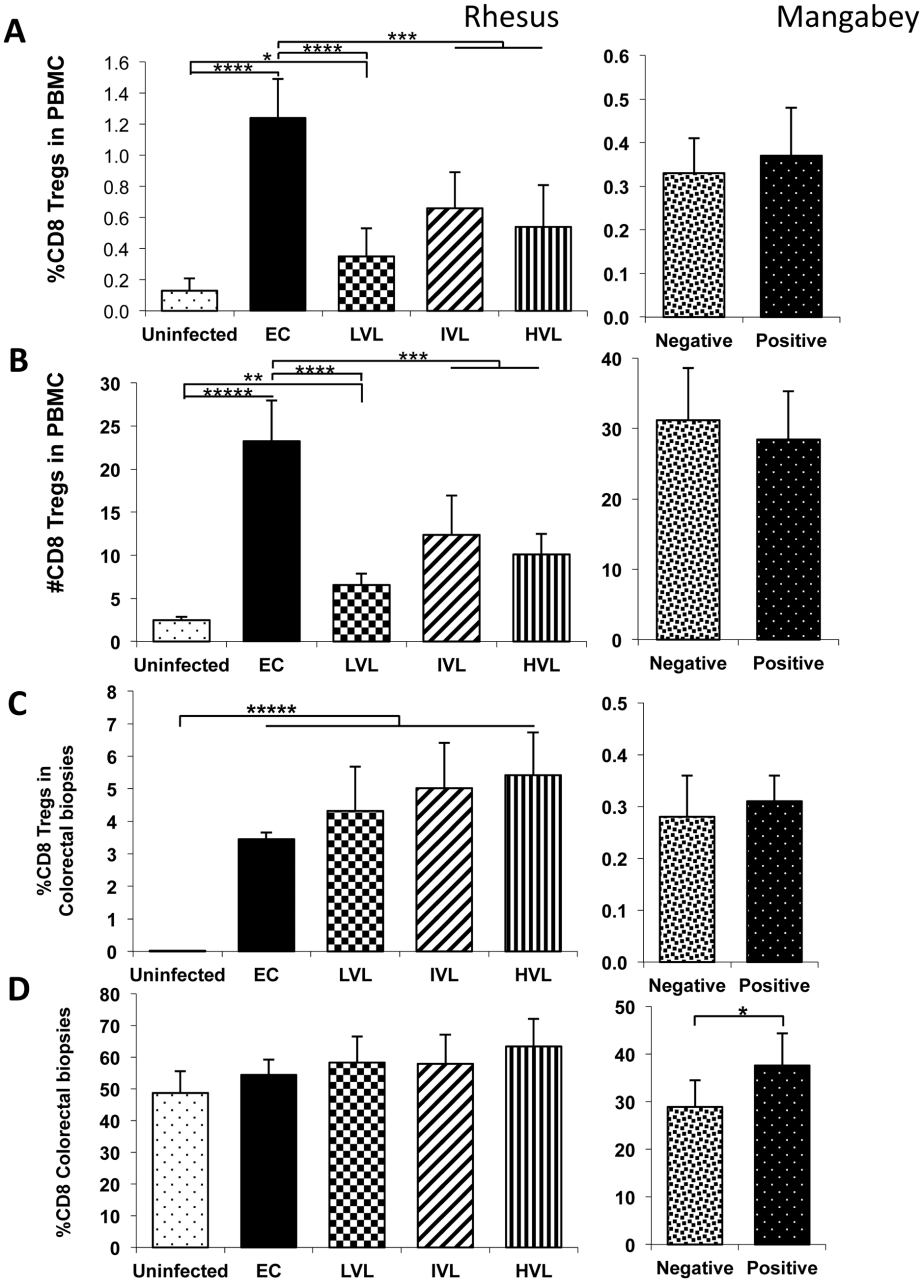
Analysis of CD8-Tregs cells in PBMC and corresponding colo-rectal biopsies from SIV infected nonhuman primates. Nomenclature identical to Fig. 1 except the data reflects values for CD8^+^ Tregs.

As seen in Fig. 6A and 6B, while there were increases in both the frequency and absolute number of CD8^+^ Tregs in the PBMC from LVL, IVL and HVL RM as compared with values in uninfected RM, there was a much more marked increase in the CD8^+^ Tregs in the PBMC from the EC RM post SIV infection (p<0.001). No increase in the frequency and absolute number of CD8^+^ Tregs was noted in the PBMC samples from SM post SIV infection.

Similar to the values seen in PBMC, there is also increase in the frequencies of CD8^+^ Tregs in the colo-rectal biopsies from EC, LVL, IVL and HVL RM post SIV infection as compared with values from uninfected RM (p<0.0001), however, the relative increases seen in the biopsy tissues seemed to be ordered as a reflection of plasma viral loads (see Fig. 6C). There were also increases in the frequencies of total CD8^+^ T cells (Fig. 6D) but except for the animals with HVL as compared with uninfected animals, these differences were not statistically significant. In addition, while there was a statistically significant increase in the frequencies of total CD8^+^ T cells in SIV infected SM, there was no significant change in the frequency of CD8^+^ Tregs.

### Reciprocal Decrease versus Increase in the Number of Plasmacytoid Dendritic Cells (pDC) in the PBMC and Colo-rectal Tissues Following SIV Infection of RM but not SM

**Figure 7:**
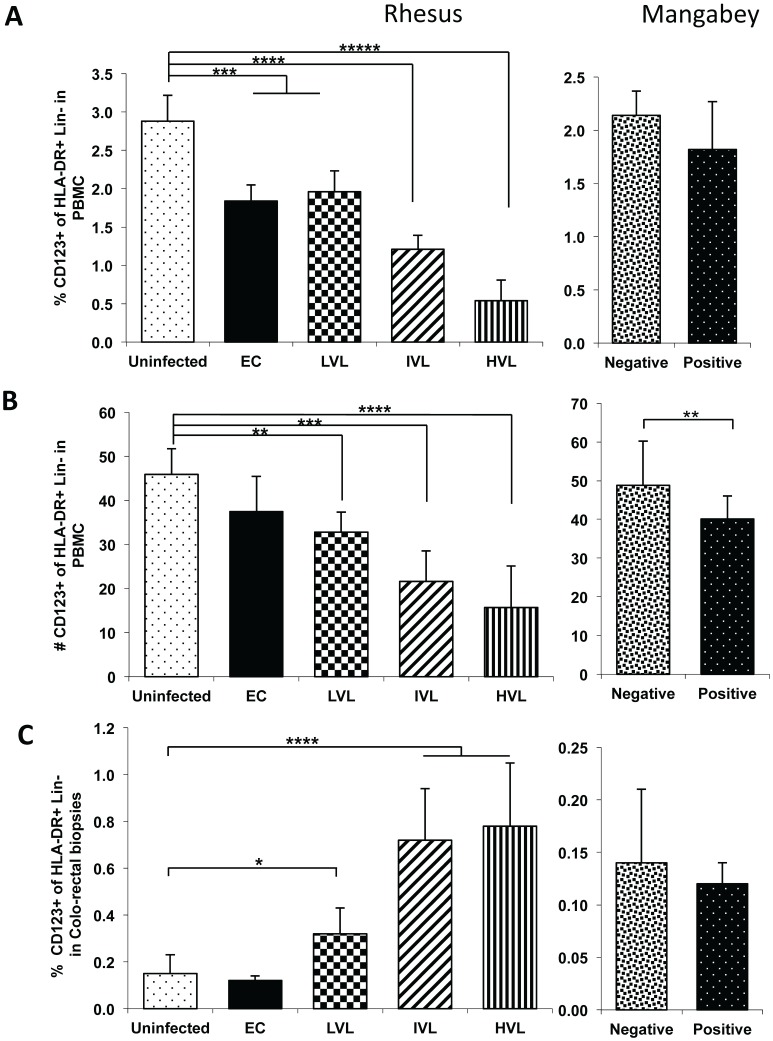
Analysis of pDCs in the PBMC and corresponding colo-rectal biopsies from SIV infected nonhuman primates. The frequencies and absolute number of CD123^+^ on the gated population of HLA-DR^+^ Lin^−^ cells (pDCs) in the PBMC and colo-rectal biopsy tissues of the same group of animals as outlined in Fig. 1. A) The frequencies of pDCs in the PBMC B) Absolute number of pDCs in the PBMC C) The frequencies of pDCs in the colo-rectal biopsy tissues from the same animals.

One of the most striking findings of this study is the highly consistent relative decrease in the frequency and absolute numbers of circulating levels of pDCs (Fig. 7A & B) and increases in the corresponding colo-rectal biopsy tissues from the same RM post SIV infection (Fig. 7C). Thus, whereas the frequencies of pDCs in the PBMC from EC and LVL monkeys decreased as compared with uninfected control RM (p<0.01), further decreases were also noted associated with IVL (p<0.001) and HVL (p<0.0001) when compared with values from uninfected RM (Fig. 7A). These differences were also reflected in the absolute numbers of pDCs in the PBMC (Fig. 7B). A small but significant decrease in the absolute numbers of pDCs in the PBMC was also noted in SIV positive SM (p<0.05). In contrast with decreases in the PBMC, the colo-rectal biopsy tissues from RM showed a marked increase in the frequencies of pDCs with increasing plasma VL except for EC monkeys (Fig. 7C). Colo-rectal biopsies from the SM, however, did not show significant changes although the values were quite variable and the numbers were significantly lower in both SIV negative and positive SM as compared with RM (please note change in Y axis, left panel versus right panel).

### Differences in the Pattern of Changes in the Frequencies of Constitutive as Compared with Induced Levels of IFN-α Synthesizing pDC in the PBMC and Colo-rectal Biopsies

**Figure 8:**
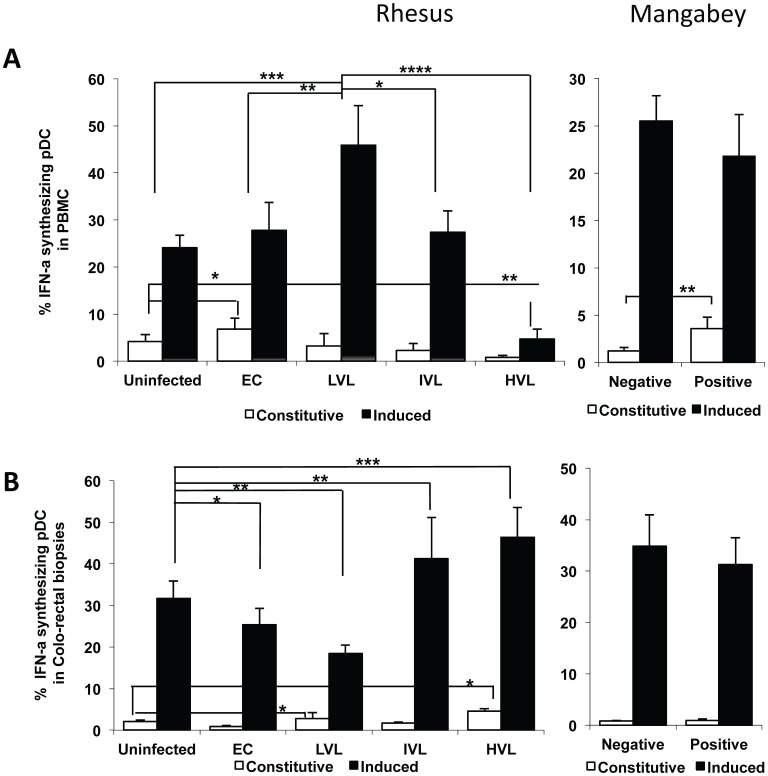
Analysis of IFN-α^+^ pDC cells in PBMC and corresponding colo-rectal biopsies from SIV infected nonhuman primates. The frequencies of IFN-α synthesizing pDCs in the PBMC (A) and colo-rectal biopsy tissues (B) of the same group of animals as depicted in Fig. 1. The blank bars reflect the frequencies of pDCs that constitutively express IFN-α and the dark bars reflect the frequencies of pDCs that are induced to express IFN-α.

A number of interesting data sets emerged when the pDCs from the PBMC and colo-rectal biopsies were examined for the frequencies that constitutively expressed IFN-α as compared with induced levels of IFN-α both in RM and SM post SIV infection. Thus, as seen in Fig. 8A, there was clearly an increase in the frequency of constitutively expressed IFN-α^+^ pDC in the PBMC from the EC (p<0.02) and a decrease in the constitutively expressed IFN-α pDC in the PBMC from the HVL RM (p<0.05) as compared with uninfected control RM. An increase in the frequency of constitutively expressed IFN-α^+^ pDC was also noted in the PBMC from SIV positive SM as compared with SIV negative SM (p<0.05). When the induced IFN-α levels were examined, the highest levels were noted in the PBMC from RM with LVL that was significantly greater than even EC RM (p<0.05). There did appear to be refractoriness to induce IFN-α with increasing viral loads as seen in HVL monkeys (p<0.001), providing support to the view that increased levels of IFN-α at least in the blood are not the basis for chronic immune activation as previously thought. Induced frequencies of IFN-α^+^ pDC varied in PBMC samples from SM and the data were therefore not significant.

The data on the frequencies of constitutive and/or induced numbers of IFN-α^+^ pDC in the cells from the colo-rectal biopsies gave a relatively straightforward pattern (see Fig. 8B). Thus, there was a low but significant increase in the frequencies of constitutive IFN-α^+^ pDC associated with progressive increases in plasma VL as compared with uninfected RM (p<0.02). In contrast, while there were significant decreases in the frequencies of induced levels of IFN-α^+^ pDC in specimens from EC RM (p<0.02) and LVL RM (p<0.05), there was a marked increase in the frequencies of induced IFN-α^+^ pDC in specimens from IVL (p<0.05) and HVL RM (p<0.01). There were no significant differences in the constitutive and induced frequencies of IFN-α**^+^** pDC in the colo-rectal tissues from SIV^+^ and SIV- SM. These data appear to suggest that increasing viral loads induce a state of refractoriness for IFN-α synthesis in pDCs from PBMC (the refractoriness was also noted in lymph node pDCs post SIV infection) and that HVL in RM is associated with increased trafficking of pDCs to the colo-rectal tissues that constitutively synthesize higher levels of IFN-α and have the capacity to chronically synthesize increasing levels of induced IFN-α. Thus, sustained high levels of IFN-α^+^ pDCs appear to be associated with HVL and poor prognosis.

### Prospective Study of pDC in the PBMC and Corresponding Colo-rectal Biopsy Tissues from RM during Acute SIV Infection

**Figure 9:**
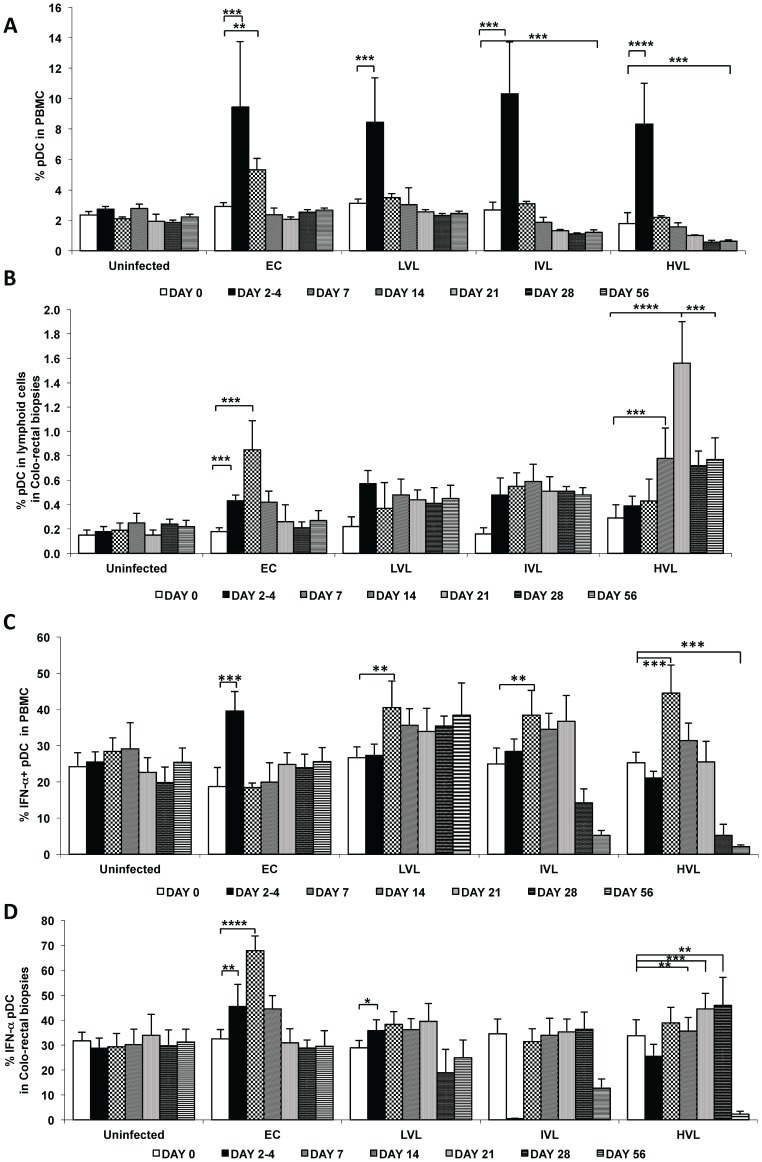
Kinetics of total and IFN-α^+^ pDCs in PBMC and colo-rectal biopsies from SIV infected macaques. The kinetics of the frequencies of A) pDCs in the PBMC, B) pDCs in the corresponding colo-rectal biopsies (C) the frequencies of the pDC shown in (A) that were IFN-α^+^ in the PBMC and D) the frequencies of pDCs that were shown in (B) that were IFN-α positive in the colo-rectal biopsy tissues. Data was obtained on a cohort of rhesus macaques prior to (Uninfected) and post intravenous infection with 1000 TCID50 of SIVmac239 collected on days 2–4, 7, 14, 21, 28 and 56 p.i. The monkeys were retrospectively classified as EC, LVL, IVL and HVL after they reached viral load set point and reached the chronic stage.

The interesting data above on marked changes in pDCs prompted us to conduct a prospective study aimed at examining the changes in the frequencies of pDCs, and the frequencies of pDCs that synthesized IFN-α and TNF-α in the PBMC and colo-rectal biopsies from the same monkey and analyze the data retrospectively after the monkeys reached viral load set point and the chronic stage so they could be classified as EC, LVL, IVL and HVL RM. The number of total cells that could be obtained from pools of biopsy tissues restricted the number of markers that could be analyzed. As seen in Fig. 9A, there is a sharp increase in the frequencies of pDCs in the PBMC very early post SIV infection (within days 2–4 p.i.) in all monkeys which gradually decreases to either baseline levels (EC and LVL) or below baseline levels (IVL and HVL). Of interest was the finding that whereas there is an increase in the frequencies of pDCs in colo-rectal tissues post SIV infection, the patterns in EC as compared with LVL, IVL and HVL are distinct (Fig. 9B). Thus, whereas there is an increase of pDCs by day 2–4 in EC, the increase was even more robust by day 7 p.i. which then rapidly decreases to baseline levels thereafter. In the case of LVL and IVL, the level of increase peaks on days 2–4 and is then sustained thereafter. However, in the case of HVL RM there appears to be a gradual increase in the frequencies of pDCs but the levels peak on day 21 p.i. (p<0.001) and then decreases but the levels remain higher then EC, LVL and IVL RM. Thus, while a transient increase in the frequency of pDCs is associated with EC, a sustained high trafficking of pDCs to the colo-rectal tissues is associated with HVL. When the fraction of pDCs in the PBMC that can induced to synthesize IFN-α was examined, once again distinct patterns emerged (Fig. 9C). Thus, whereas there was a transient increase in the IFN-α^+^pDCs in the PBMC from EC, an increase also occurred in the PBMC from LVL but this increased potential was sustained in this group of LVL animals. On the other hand, the increase in the frequency of IFN-α^+^ pDC in the PBMC was followed by a gradual decrease in both the IVL and HVL RM with a more marked decrease in HVL group. These data suggest that low levels of viremia must sustain increased potential of pDCs in the PBMC to synthesize IFN-α but that with increasing VL this function is compromised even though there is marked decrease in the number of pDCs in HVL RM during chronic infection (Fig. 7A). When the induced frequencies of IFN-α synthesizing pDCs from the colo-rectal biopsy tissues were examined, there appeared to be a sharp increase in the levels on day 2–4 (p<0.05) and day 7 p.i. (p<0.001) in samples from EC monkeys which subsequently returned to baseline levels (Fig. 9D). While there were some changes in the frequencies of IFN-α synthesizing pDCs in samples from LVL and IVL monkeys, the samples from the HVL monkeys showed a pronounced sustained increase during the acute infection period up to day 56 but it is important to note that there is a marked reduction in IFN-α synthesizing pDCs during late chronic infection (>36 weeks p.i.). These data suggest that a transient increase in IFN-α^+^ pDCs is associated with EC but a sustained increase during acute infection is associated with HVL, which subsequently becomes refractory to IFN-α synthesis.

### Marked Increases in TNF-α Synthesizing pDCs is a Valuable Marker for SIV Infected RM with IVL and HVL and Absence of TNF-α^+^ pDC is an Important Marker for EC RM

When the frequencies of TNF-α synthesizing pDCs in the PBMC and colo-rectal biopsy tissues were examined, the levels in the PBMC did not show any significant change in the various groups of monkeys (data not shown). However, in the case of the colo-rectal biopsy tissue obtained cells, there was a clear association of a gradual increase in TNF-α^+^ pDCs selectively in monkeys with IVL and even more pronounced increase in monkeys with HVL (see supplementary Figure S3) but not in monkeys classified either as EC or those with LVL. Of interest, these increases in the IVL were noted by day 14 (p<0.05), with further increases by day 28 (p<0.01) and day 56 (p<0.001). The increases in HVL monkeys were seen in samples post day 21 (p<0.05), day 28 and day 56 (p<0.0001). These data suggest that lack of an increase in TNF-α^+^ pDCs is associated with EC and that the appearance of TNF-α synthesizing pDCs is associated with increasing viral loads as seen in monkeys with IVL to HVL.

## Discussion

The interaction between viruses, viral genomes, viral proteins, and the various intermediates of viral replication products with host pathogen recognition receptors (PRRs) is the initiating event that play a deterministic role in the quality and quantity of the host response that is generated against viral pathogens. Such initial interactions leads to the synthesis and the release of a wide variety of mediators which include cytokines, chemokines, and interferons (IFN) and the modulation of a variety of cell surface molecules expressed by the interacting cells [37]–[39]. Under physiological conditions, this constellation of cells must assimilate and integrate such diverse signaling events during acute infection and integrate the biological dialog occurring at the cell membrane and that occurring within the cell into an efficient orchestrated function which is in the correct temporal and spatial environment to achieve a biologically functional state that is of benefit to the host and maintains homeostasis. Unregulated signaling via any of these pathways during acute infection are undesirable outcomes for the host and result in events such as “cytokine storms” accompanied by injury promoting inflammation, tissue damage and in select cases lethal to the host [40], [41]. Therefore, the net results of such interactions during acute infection is determined by the balance between the pro-inflammatory and the anti-inflammatory mechanisms which sets the foundation for the physiological generation of acquired immune responses that have the potential for clearance of the virus. Dysregulation of these events, on the other hand leads to ineffective acquired immune responses and of disease consequences for the host. A detailed study of this interface between the innate and acquired immune effector cells and molecules in HIV/SIV infection has been lacking. The purpose of the studies reported herein was to begin to systematically address this gap in our knowledge.

In the present study, the availability of a large cohort of rhesus macaques infected with SIVmac239 or 251 that displayed a wide range of viral loads during chronic infection were utilized to examine the profile of IL-17 synthesizing cell lineages including CD4, CD8 and subsets of NK cells and other cell lineages such as CD4^+^ and CD8^+^ Tregs that have been reasoned to have an impact on the IL-17 synthesizing cells, particularly within the gastrointestinal tissues. Finally, studies also included the delineation of the frequencies of pDCs that synthesize IFN-α.

The importance of the cytokine IL-17 and the cell lineages that synthesize IL-17 in particular the Th17 cells has been highlighted due to their ability to synthesize IL-22 and thus a role in maintaining GIT integrity and its potential role in the pathogenesis of both human and nonhuman primate HIV/SIV infections [22], [24], [25], [28]. Overall, the results of the studies reported herein show that there is a marked decrease in both the frequencies and absolute numbers of IL-17 synthesizing CD4^+^ Th17 cells in the blood and colo-rectal tissues associated with increasing viral loads (Fig. 1A & B). Of interest was the finding that there is also a decrease in the absolute number of this same lineage in SIV^+^ mangabeys in the blood with a trend also in the GI tissues (Fig. 1 C & D). At face value these data suggest that this decrease is related to viremia and not disease associated although one could argue whether the finding in the mangabeys are relevant to rhesus macaques. What was interesting to note that in contrast to the data on CD4-Th17, the absolute number of Tc-17^+^ cells in the blood increased (Fig. 2B) but decreased in frequencies in the colorectal tissues (Fig. 2C). These data suggest that the mechanisms associated with regulation of IL-17 synthesizing cells in the blood versus the GI tissues must be different either qualitatively and/or quantitatively. Similarly, while the frequencies of NK-17 also decreased in the blood and GI tissues of rhesus macaques that correlated with plasma VL, the absolute numbers in the blood and by inference the GI tissues in fact increased (Fig. 3). The recent finding that the IL-21 synthesizing CD4^+^, CD95^+^, CCR6^−^ memory CD4^+^ T cells plays such a regulatory function [42] prompted us to analyze the frequencies of this cell lineage in a subset of these same monkeys. Indeed, SIV infection leads to depletion of this subset (data not shown) that correlates well with the frequencies of CD4-Th17 in the blood and GI tissues but not Tc17^+^ and NK-17^+^ cells in the blood. This imbalance in the ratio of Th17 and Tc17 is consistent with previous reports by Kader et al [43]. It is also important to note that SIV infection also leads to decreases in the frequencies and absolute numbers of Tc17^+^ in the mangabey although the numbers of IL-21^+^ CD4^+^ T cells did not change in this species. These data suggest that there may be different mechanisms for the regulation of IL-17 synthesizing CD4 as compared with Tc17 cells and NK-17 cells, a matter of current study. The increased absolute numbers of NK-17^+^ cells in the blood of monkeys with increasing plasma VL also appeared to be highly activated suggesting that such cells could be attempting to regulate anti-viral CD8^+^ T effector cell function as described elsewhere [44] promoting chronic infection.

During the course of analyzing the frequencies of IL-17 synthesizing cells, it was noted that there were additional subsets of the NK cell lineage uniquely present in the GI tissues that expressed IL-17 that prompted us to include these cell lineages in our analysis. This includes the CD3^−^, CD8^+^, NKG2a^−^ cells which most likely include the NKp44^+^ cells [30] and the previously not described CD3^+^, CD8^+^, NKG2a^+^ cell lineages. There were marked decreases in the frequencies of both of these cell lineages (Fig. 4B to D) and the fraction of which were induced to express IL-17 (Fig. 4A & C). Whether these 2 cell lineages are under the regulation of the CD4^+^ IL-21 synthesizing cells is not clear at present but is consistent with a loss of this subset in the GI tissues as described above. These data also suggest plasticity in the role of IL-17 synthesizing cell lineages [45]–[47]. In addition, it is important to note that not all Tc17 cells express granzymes and/or killer function but a high frequency express the co-inhibitory molecule CTLA-4 [27] and thus changes in their levels need to be interpreted with these functional attributes in mind.

The connective and interplaying role of CD4-Tregs with the Th17 lineage of cells in particular, has been highlighted by a number of studies and reviewed recently [48], [49]. The general consensus is that these CD4-Tregs suppress anti-viral T cell response but not T cell activation. More recent data shows that the target gene for FoxP3 the hallmark transcription factor that identifies nTregs, is the promoter region of IL-22 [50] suggesting that the activation of CD4-Tregs leads to down regulation of IL-22 synthesis by Th17 cells, thus dialing down the inflammatory role of Th17^+^ cells. What is difficult to understand within this context is that while IL-22 is required to maintain GI tissue integrity, increased Treg function would lead to decreased Th17 induced IL-22 production. Clearly more studies are needed to better define the interplay and it is perhaps the kinetics by which such interplays are manifest that dictate the results. In the studies reported herein, the data are very consistent with those summarized by Hartigan-O’Connor et al [48]. Thus, there clearly is a significant increase in the frequency and absolute number of CD4-Tregs (Fig. 5A to D) in both the blood and colo-rectal tissues in the EC rhesus macaques but marked decreases associated with increasing VL in the rest. A somewhat different picture emerged with the analysis of CD8-Tregs. Thus, there were increases both in the frequency and absolute numbers of CD8-Tregs in the blood of EC, LVL, IVL and HVL as compared with uninfected control animals (Fig. 6A & B). The increases were most marked in EC’s. These data are basically in agreement with those published earlier by Nigam et al [51] and suggests that increased levels are associated with increased viral loads and poor prognosis. However, the increased levels seen in EC monkeys particularly in the blood needs address. While CD8^−^Tregs were in fact described a long time ago and coined suppressor T cells, the data has accumulated since that immune regulation is in fact quite complex and comes in multiple flavors. Thus, there are natural Tregs (nTregs), IL-10 producing Tregs (Tr1), TGF-β producing Tregs (Th3), CD8-Tregs, more recently even NK-T regs and NK-regs [52]–[54]. The CD8-Tregs described herein are the classical FoxP3 expressing regulatory CD8^+^ T cells that also express CTLA-4 and CD39. Whether the CD8-Tregs in EC are functionally similar to the CD8-Tregs in monkeys with HVL is not clear at present although preliminary studies indicate that they neither express granzymes and are not likely to be cytolytic. Studies in progress indicate that the microarray profiles of CD8-Tregs from EC are quite distinct from CD8^+^Tregs from HVL monkeys and are currently being studied. These findings highlight the concept of split personality of such regulatory T cells recently reviewed [55].

There has been increased interest on the role of the pDCs as playing a major contributory role in dictating the outcomes of HIV/SIV infection [26], [33], [56]–[60]. This concept has been driven by the finding that the pDCs are the major source of IFN-α [61] that mediates potent anti-viral effects particularly during acute infection and hence the importance of this cell lineage. Our lab was the first to document the finding of a rapid mobilization (within 2–4 days p.i.) of pDCs in the blood followed by its accumulation within the GI tissues of SIV infected rhesus macaques [26]. However, a detailed study of its relationship with plasma VL was not studied. Thus, it was reasoned that a study of this cell lineage and its role in the IL-17/Treg/pDC axis important. As noted above, there was a very good relationship between levels of plasma viremia and frequencies and absolute numbers of pDCs both in the blood and colo-rectal tissues (Fig. 7 A to C). Thus, increasing VL led to decreasing levels of pDCs in the blood but of interest increasing levels in the corresponding colo-rectal tissues. We next determined the frequencies of constitutive versus induced frequencies of IFN-α synthesizing pDCs. The most striking change noted was the high frequencies of IFN-α synthesizing pDCs in LVL versus HVL with IVL showing intermediate levels in the blood (Fig. 8 A) and the opposite in the colo-rectal tissues (Fig. 8B). These data seem to imply that while the number of pDCs in HVL decrease in the blood, they are even further reduced in their ability to synthesize IFN-α but those homing to the gut increase both in number and their ability to synthesize IFN-α suggesting that a sustained IFN-α responsive pDCs has poor prognosis which is an important finding with implications for vaccines that are being engineered to induce potent pDCs activation specially during acute infection. This is further emphasized by the findings that lower frequencies of pDCs and relatively lower levels of IFN-α appear to be associated with EC and LVL. In sum, these data seem to suggest that a well regulated approach to mobilize a small number of pDCs may be beneficial to the host.

The increased importance of pDCs documented in a number of studies in HIV-1 infected EC [59] including their differential role in natural SIV disease resistant and non-natural disease susceptible hosts [26] prompted us to initiate a more detailed prospective study of pDCs. What was of interest was the finding of a marked increase in the number of pDCs in the blood of all animals regardless of the eventual VL (Fig. 9A). Thus this increased mobilization must be a general function of viral infection and does not predict future plasma VL. Within this context, the difference in the profile of LVL as compared to HVL was of interest. Thus, while the increase seen during early p.i. is maintained to the same level in the LVL, there is a further marked increase in the HVL peaking at day 21 p.i. and the increased frequency is maintained at the level seen at day 14 in these HVL monkeys. These data appear to suggest that increased levels of pDCs in the GI tissues is of predictive value that is destined to result in HVL. An initial increase followed by stabilization was clearly optimal since it resulted in EC. However, a sustained moderate increase was associated with higher VL. Eventually, however, the pDCs even in the HVL become refractory to IFN-α induction during late chronic infection suggesting that there is a lack of a contributory role of IFN-α to chronic immune activation, an issue that has been addressed in several previous studies [62]–[64]. The reasons for these differences are not clear at present, although an important role of IFN-α has been documented in HIV-1 infection [37], [65]–[67]. In addition, to investigate this issue further, we also analyzed the blood and colo-rectal tissue for select ISGs. What was interesting was the finding that in both blood and colo-rectal tissues an initial rise followed by a marked diminution of the levels of these ISGs was associated with EC and LVL but high sustained levels of ISGs at the mRNA level were associated with IVL and HVL (Figure S4). These data suggest that interferon stimulating genes other than IFN-α may be contributing to chronic immune activation, a subject of current study.

In summary, therefore, with a focus on parameters in the GI tissues, a decrease in a variety of IL-17 synthesizing cell lineages including CD4^+^ T cells (Th17), Tc17 cells, NK-17 cells and the CD3^−/^CD8^+^/NKG2a^−^ NK cell subset in association with decreased frequencies of CD4-Tregs but increased CD8-Tregs and pDCs with sustained IFN-α synthesizing pDCs (including increased levels of sustained ISGs) predicts HVL and poor clinical outcome. The appearance of an increase in the frequencies of pDCs that synthesize TNF-α at the end of the acute infection period, similarly, has poor prognostic outcome. In contrast, maintenance of high levels of IL-17 synthesizing cell lineages coupled with increased levels of CD4^+^ Tregs but maintenance of a normal frequency of CD8-Tregs, and lower levels of pDC with moderate ability to synthesize IFN-α and lower levels of ISGs and TNF-α synthesizing pDCs are associated with EC and to some extent LVL. Thus, for vaccine design these paradigms should provide an important initial foundation and are being tested in candidate vaccine formulations at our institution and elsewhere. It is also clear from these studies that pDCs become refractory to TLR induced IFN-α synthesis despite sustained increases of ISG’s providing suggestive evidence that genes other than those encoding IFN-α are likely contributing to chronic immune activation. Finally, while initially perhaps the ratio of CD4^+^Tregs:Th17 maybe critical such as seen in EC and LVL, shortly thereafter it seems that the virus depletes the Th17 and thus CD4^+^Tregs cannot manifest their function since their targets which are Th17 are depleted by direct virus effects. Thereafter, it is the CD8^+^Tregs that seem to play a major negative role particularly in the GI tissues. These findings provide a number of novel targets for immune mediate modulation.

## Supporting Information

Figure S1 Summary of plasma viral load outcome of SIV infection of rhesus macaques. Classification of 103 rhesus macaques infected intravenously with either SIVmac239 or 251 based on plasma viral loads after reaching VL set point and during chronic infection. They were classified either as monkeys with >10^6^ viral copies/ml (HVL), those with 250,000 to 1 million viral copies/ml and those with 10,000 to 250,000 viral copies/ml (IVL), those with <10,000 viral copies/ml (LVL) and those with undetectable viral loads (EC).
(TIFF)

Figure S2 Representative profile of the gating strategies utilized for defining the frequencies and absolute numbers of A) CD4^+^-Th17, CD8^+^ Tc17, CD3^−^,CD8^+^, NKG2a^+^-NK17 cells and B) IFN-α synthesizing plasmacytoid dendritic in the PBMC of rhesus macaques.
(TIFF)

Figure S3 Kinetics of TNF-α^+^ pDCs in PBMC and colo-rectal biopsies from SIV infected macaques. Frequencies of plasmacytoid dendritic cells (pDCs) from colo-rectal biopsy tissues that expressed TNF-α from a cohort of rhesus macaques infected intravenously with 1000 TCID50 of SIVmac239. Samples from the same monkeys were collected prior to (Day 0), and on days 7, 14, 21, 28, and 56 p.i. The total number of pDC in these samples are reflected under Fig. 9B in the text.
(TIFF)

Figure S4 Kinetics of the expression of interferon stimulating genes (ISG’s) in colo-rectal biopsies of SIV infected macaques. Kinetics of the expression of (A) 2, 5 oligoadenylate synthetase (OAS) and (B) myxovirus resistance protein A (MxA) at the mRNA level in aliquots of colo-rectal biopsies of a cohort of rhesus macaques prior to (Uninfected) and post intravenous infection with 1000 TCID50 of SIVmac239. Data reflect the fold change in the levels of mRNA on specimens collected on day 7, 14, 21, 56 and 84 p.i. as outlined in the Methods section.
(TIFF)
