# Supplementary figures and images for: Relationships between IL-17+ Subsets, Tregs and pDCs That Distinguish among SIV Infected Elite Controllers, Low, Medium and High Viral Load Rhesus Macaques
Source: PLoS One. 2013 Apr 19;8(4):e61264. doi: 10.1371/journal.pone.0061264 (PMC3631185; doi:10.1371/journal.pone.0061264)

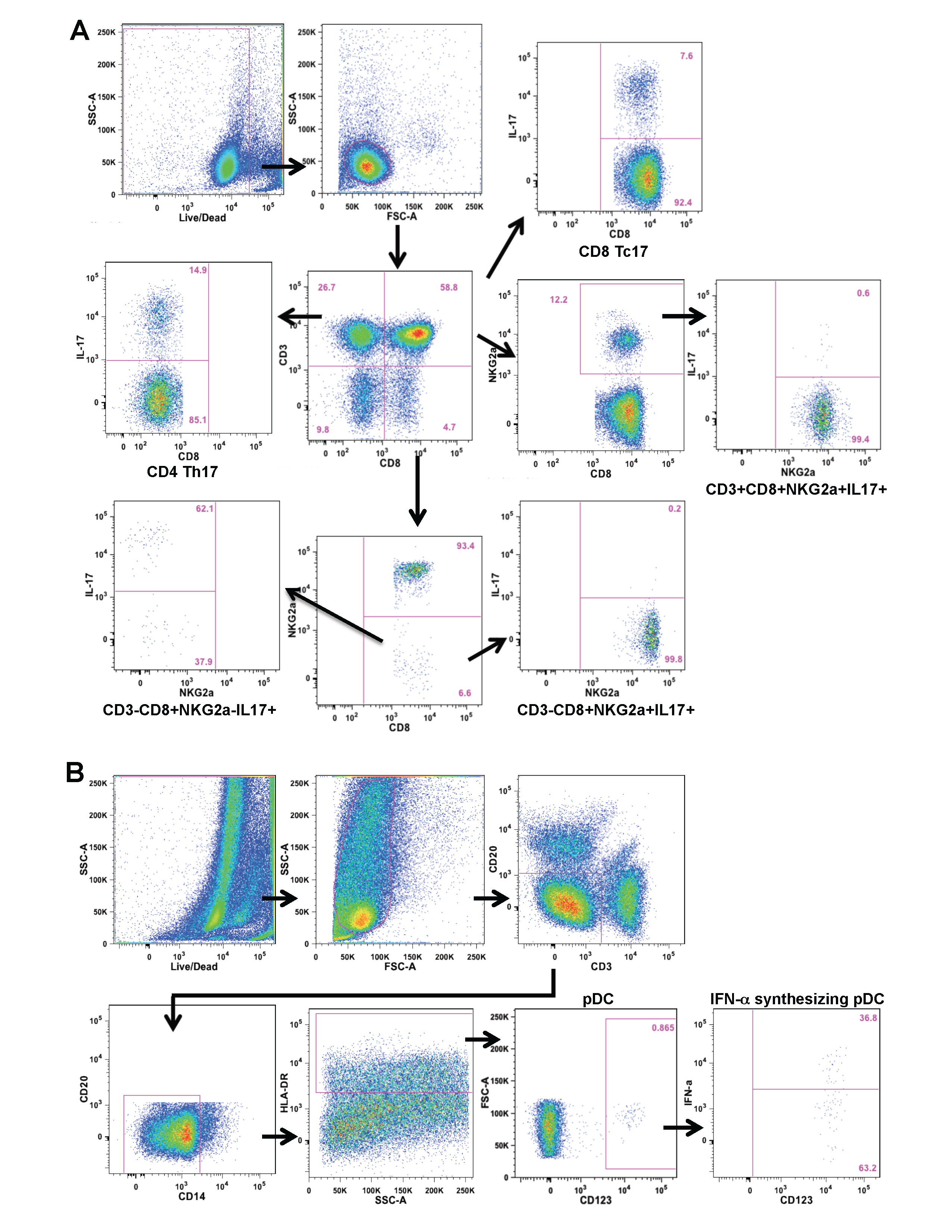

Supplement: Figure S2 — Representative profile of the gating strategies utilized for defining the frequencies and absolute numbers of A) CD4+-Th17, CD8+ Tc17, CD3−,CD8+, NKG2a+-NK17 cells and B) IFN-α synthesizing plasmacytoid dendritic in the PBMC of rhesus macaques. (TIFF) [file pone.0061264.s002.tif]

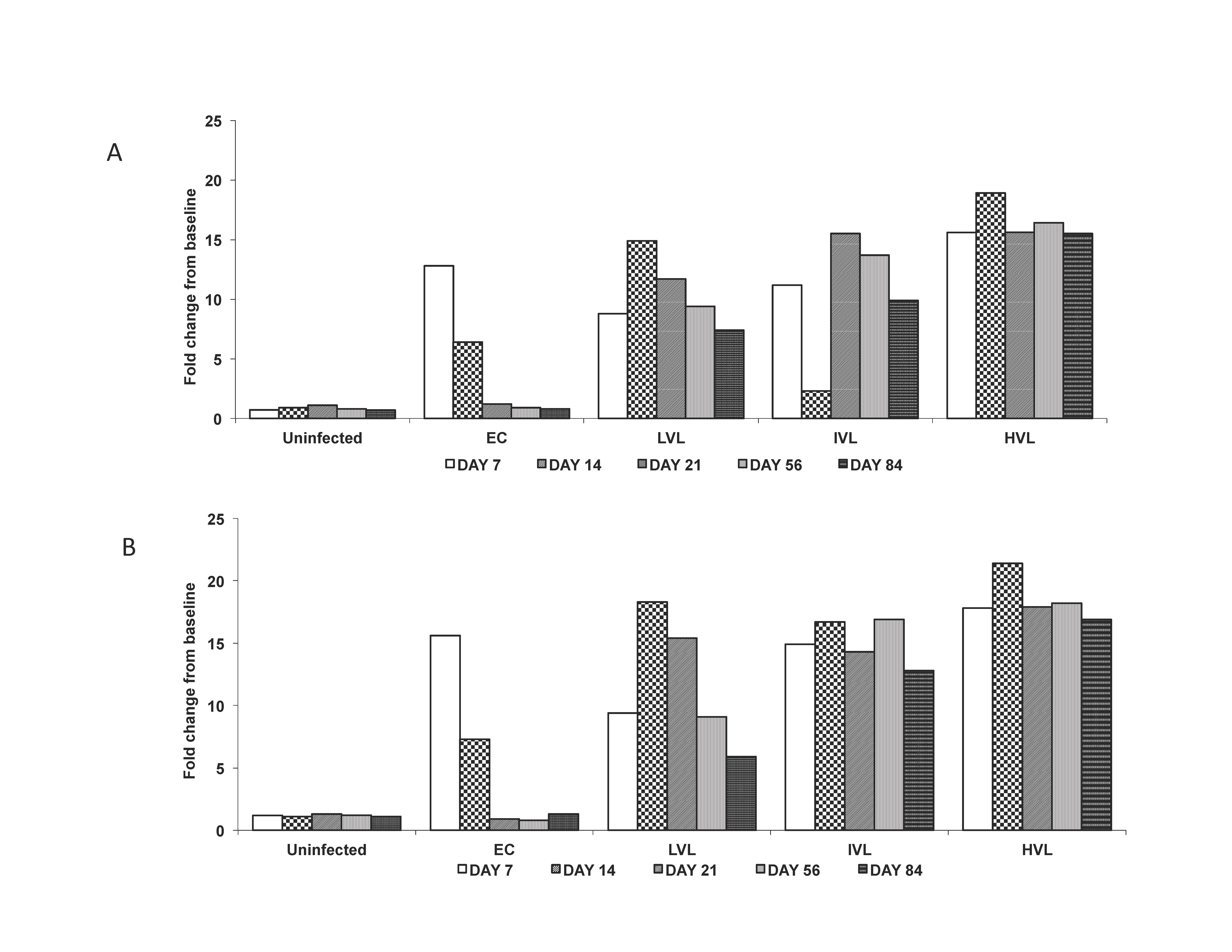

Supplement: Figure S4 — Kinetics of the expression of interferon stimulating genes (ISG’s) in colo-rectal biopsies of SIV infected macaques. Kinetics of the expression of (A) 2, 5 oligoadenylate synthetase (OAS) and (B) myxovirus resistance protein A (MxA) at the mRNA level in aliquots of colo-rectal biopsies of a cohort of rhesus macaques prior to (Uninfected) and post intravenous infection with 1000 TCID50 of SIVmac239. Data reflect the fold change in the levels of mRNA on specimens collected on day 7, 14, 21, 56 and 84 p.i. as outlined in the Methods section. (TIFF) [file pone.0061264.s004.tif]
